# Histologic, immunohistochemical, and molecular features of pituicytomas and atypical pituicytomas

**DOI:** 10.1186/s40478-019-0722-6

**Published:** 2019-05-02

**Authors:** Angela N. Viaene, Edward B. Lee, Jason N. Rosenbaum, Ilya M. Nasrallah, MacLean P. Nasrallah

**Affiliations:** 1 Department of Pathology and Laboratory Medicine, Children’s Hospital of Philadelphia, University of Pennsylvania Perelman School of Medicine, Philadelphia, PA USA; 2 Department of Pathology and Laboratory Medicine, University of Pennsylvania Perelman School of Medicine, Philadelphia, PA USA; 3 Department of Radiology, University of Pennsylvania Perelman School of Medicine, Philadelphia, PA USA; 4 Hospital of the University of Pennsylvania, FO6.089 3400 Spruce St, Philadelphia, PA 19104 USA

**Keywords:** Pituicytoma, HRAS, BRAF, Neurohypophysis, Sella, MAPK pathway

## Abstract

Pituicytoma is a rare, poorly characterized tumor of the sellar region that is thought to be derived from neurohypophyseal pituicytes. Resection of pituicytomas is often associated with significant morbidity including diabetes insipidus and panhypopituitarism. Most of the literature on this tumor exists as small case series or case reports. Here we describe a cohort of fourteen pituicytoma resections from eleven patients. The average follow-up on these cases is 3.7 years with some patients having over 10 years of follow-up data available in the electronic medical record. Pituicytomas were frequently misdiagnosed on pre-operative imaging, and surgical resection was associated with persistent endocrine abnormalities. Histologically, the tumors showed a range of morphologies from epithelioid to spindled. All tumors were positive for TTF-1 with variable immunostaining for other markers including GFAP, EMA, S100, SSTR2A, and synaptophysin. Within this cohort are two patients with atypical pituicytomas which showed increased cellularity, pleomorphism, mitoses and elevated Ki-67 proliferation indexes when compared to non-atypical pituicytomas. Next generation sequencing performed on three tumors revealed alterations in genes involved in the MAPK pathway. Additionally, immunohistochemical staining for phosphorylated-ERK was positive in the majority of tumors. Increased awareness of the neoplastic entity and identification of targetable mutations have the potential to decrease the morbidity associated with resection of pituicytomas.

**Electronic supplementary material:**

The online version of this article (10.1186/s40478-019-0722-6) contains supplementary material, which is available to authorized users.

## Introduction

Pituicytoma is a rare, poorly characterized tumor of the sella and suprasellar region that is thought to be derived from neurohypophyseal pituicytes and thus stains positively for thyroid transcription factor 1 (TTF-1) [11]. The initial characterization of the tumor was performed in a series published in 2000, in which all pituicytomas were found to be low grade and recurred only if incompletely resected [1]. Following the publication of this article, pituicytoma became an official WHO entity and is now listed in both the WHO Classifications of Tumours of Endocrine Organs and WHO Classifications of Tumours of the Central Nervous System [13, 14] where it is designated to correspond histologically to a WHO grade I tumor. Despite its generally low grade features, patients often present with visual disturbance, headache and endocrine abnormalities necessitating surgical resection [3, 6]. Resection of pituicytoma has been associated with significant morbidity, including diabetes insipidus and panhypopituitarism [6, 8, 27, 28], which have life-altering negative consequences for patients.

Due to the rare nature of pituicytomas, most of the literature on this tumor exists as small case series or case reports. While most pituicytomas have been found to be low grade and indolent, atypical pituicytomas have been reported [11]. Histologically, pituicytomas have been shown to have a range of morphologies [1, 7, 14, 19, 23, 24]. Positive staining for TTF-1 is a common feature [11]. Additionally, spindle cell oncocytomas and granular cell tumors of the sellar region have also been shown to be positive for TTF-1 [11, 15] suggesting a common pituicyte origin. It has been proposed that spindle cell oncocytomas and granular cell tumors of the sellar region are morphologic variants of pituicytoma [10, 11, 15]. This notion is supported by the existence of five subtypes of pituicytes within the neurohypophysis including oncocytic, granular and “major” (astrocyte-reminiscent) pituicytes [22], potentially leading to the somewhat varied morphologies amongst these tumors.

As molecular testing has become an increasingly integral part of surgical pathology, recently a few attempts have been made to elucidate molecular signatures of pituicytomas. These tumors have been found to be negative for *IDH* variants, *BRAF* variants and *BRAF* fusions, which are changes commonly found in low grade brain tumors [15]. One case has been studied with array-based comparative genomic hybridization, and the tumor demonstrated genomic copy number imbalances with losses of chromosome arms 1p, 14q, and 22q, and overrepresentation of chromosome arm 5p [18]. In contrast, whole exome sequencing performed on three patients’ pituitary spindle cell oncocytomas, which again may be a variant of pituicytoma, yielded no copy number imbalances, but did find the activating mutation in *HRAS* c.182A > G p.(Q61R) [16]. *HRAS* encodes a protein involved in the mitogen-activated protein kinase (MAPK) pathway, which regulates cell division in response to growth factors, and the gene is known to be mutated in cancers.

Here we present a cohort of eleven patients with pituicytomas, including two atypical pituicytomas, and describe the clinical, radiologic, histologic, immunohistochemical and molecular findings of these tumors. We further propose that alterations in the MAPK signaling pathway lead to changes in the pathway that could be detectable at the protein level by immunohistochemical techniques. The delineation of this disease-associated mutation in pituicytomas may allow the future use of targeted therapy in the treatment of this tumor.

## Materials and methods

This study was approved by an independent institutional review board at the Hospital of the University of Pennsylvania (HUP IRB 827290). A database search was performed for all pathology specimens from January 1, 1990 through June 30, 2018 to identify surgically resected pituicytomas, spindle cell oncocytomas, and granular cell tumors of the sellar region. All identified cases were then reviewed by two neuropathologists board-certified by the American Board of Pathology (ANV and MPN) to confirm the histologic diagnosis on permanent sections. When necessary, an additional immunohistochemical stain (TTF-1) was performed (see immunohistochemical staining methods below).

### Immunohistochemistry

Immunohistochemical studies performed as part of the initial diagnostic workup were reviewed and included in this study. An additional TTF-1 stain was performed on one case to confirm the diagnosis of pituicytoma. Staining for Somatostatin Receptor 2 (SSTR2A), BRAF V600E and phosphorylated-ERK (pERK) were performed as part of this research study. For each tumor, the number of Ki-67 positive cells was estimated by two neuropathologists board-certified by the American Board of Pathology (ANV and MPN). When calculating the percentage of tumors positive for an immunostain, focal positivity was counted as positive. Additionally, results for patient #10 were summarized across all four resections to give a single result for each marker, and therefore this patient was only counted once in percent-positive calculations. Five non-neoplastic pituitary glands containing both anterior and posterior pituitary (all autopsy specimens) were also stained with pERK.

TTF-1 (Leica, PA0364), EMA (Dako, M0613, 1:100), S100 (Dako, IR50461), GFAP (Dako, M0761, 1:400 with Leica AR9352), synaptophysin (Cell Marque, 336-R-98), Ki-67 (Dako, IR62661), SSTR2A (Abcam, ab134152, 1:100), BRAF V600E (Ventana 790–4855, clone VE1) and pERK (Cell Signaling, 4370) antibodies were used to stain formalin fixed paraffin embedded slides. Staining was performed on the Leica Bond-IIITM Autostainer using the Bond Polymer Refine Detection System (Leica Microsystems DS9800) with the DAB chromagen. For GFAP and pERK, 20 min of heat-induced epitope retrieval with EDTA buffer, pH 9.0 (Leica Microsytems) preceded staining. For Ki-67 and SSTR2A, 20 min of heat-induced epitope retrieval with citrate buffer, pH 6.0 (Leica Microsystems) preceded staining. Nuclei were counterstained with hematoxylin.

### Next generation sequencing

Next generation sequencing (NGS) was performed on two pituicytomas at the request of the patients’ clinical team. Testing was performed at the Center for Personalized Diagnostics (CPD) at the University of Pennsylvania, a CLIA-approved laboratory. Genomic DNA was extracted from formalin-fixed paraffin-embedded tissues and run on the Solid Tumor Panel for both tumors. Additionally, RNA was extracted and run on the Fusion Transcript Panel for one of these tumors. The Solid Tumor Panel uses a custom Agilent HaloPlex library preparation (Agilent, Santa Clara, CA) to cover approximately 0.5 megabases, including the entire exonic (coding) sequence of 152 genes, + 10 base pairs of intronic sequence. The library preparation includes unique molecular identifiers to identify duplicate reads. Specimens were sequenced on the Illumina HiSeq 2500 platform (Illumina, San Diego, CA) using multiplexed, paired end reads. Analysis and interpretation was performed using a customized bioinformatics pipeline, Halo_v1.2. All variants were annotated with reference to the hg19 Genome build. Variants are reported according to HGVS nomenclature and classified into 3 categories: Disease-Associated Variants, Variants of Uncertain Significance, and Benign. Variant allele fraction (VAF) is defined as the number of reads of a variant from the reference sequence divided by the total number of reads at that position. The Fusion Transcript Panel uses an anchored multiplex PCR strategy to sequence from RNA template, targeting specific exons, identifying oncogenic transcripts agnostic of the specific fusion partner or exon skipping event [21]. Fusion transcripts, oncogenic splice-forms, and large deletions can be detected. The full list of the 152 genes sequenced on the CPD Solid Tumor Panel and the 55 genes tested on the Fusion Transcript Panel can be found at (https://www.pennmedicine.org/departments-and-centers/center-for-personalized-diagnostics/gene-panels).

As part of this research study, sequencing on the CPD Solid Tumor Sequencing Panel was attempted for an additional eight tumors using the above techniques.

### Radiologic diagnostics

Ten of the eleven patients with pituicytoma had pre-operative MRI studies available. A neuroradiologist (IMN) blinded to the diagnoses reviewed the imaging studies, as well as interspersed studies from seven patients with pathology-proven pituitary adenomas and three patients with pathology-proven craniopharyngiomas. The radiologist was asked to give a single diagnosis per patient, given the knowledge that half of the patients had pituicytomas. The non-pituicytoma cohort was matched for age, sex and year of resection to the pituicytoma cohort.

## Results

### Patient characteristics

The database search identified fourteen pituicytoma specimens, zero spindle cell oncocytomas, and zero granular cell tumors of the sellar region. These fourteen pituicytoma specimens were found to be from eleven patients (one patient had four separate resections), and the clinical findings of these patients are detailed in Table 1. Three patients in this study (#9, #10 and #11) were included in a previous publication (Lee et al., 2009, cases 3, 4, and 5, respectively [11]). The male to female ratio was 1.2:1, and the average patient age at the time of initial surgery was 53 ± 16 years (mean ± standard deviation; range 30–77 years). The initial presenting symptoms varied across patients; 8 (73%) presented with visual disturbances and/or abnormal hormone levels, 6 (55%) with visual symptoms and 5 (45%) with hormone imbalances. Three patients (27%) were diagnosed incidentally on imaging for other conditions. For patients followed within our hospital system, the average follow-up was 3.7 ± 3.3 years (range 0.5–10.1 years). Two patients were deceased secondary to complications of surgery or treatment (#9 and #10). Patient #9 suffered cardiopulmonary arrest immediately following surgery and was unable to be resuscitated. Patient #10’s tumor recurred multiple times. She received both chemotherapy and radiation and ultimately developed carotid occlusion with multifocal brain infarcts thought to be secondary to radiation therapy. All surviving patients were found to have panhypopititarism and/or diabetes insipidus following pituicytoma resection.

**Table 1:** Clinical and radiologic characteristics of pituicytomas

Patient	Gender	Age at diagnosis (years)	Visual Symptoms	Hormonal Symptoms	Incidental finding	Size on imaging (cm)	Sellar	Supra-sellar	% of tumor resected	Follow-up (years)	Progression/Recurrence	Panhypopit-uitarism/DI	Deceased
1	M	47	N	Y	N	3.1 (MRI)	Y	Y	95%	8.4	N	Y	N
2	F	41	Y	Y	N	1.9 (MRI)	Y	Y	–	1.2	N	Y	N
3	M	64	Y	N	N	3.5 (MRI)	Y	Y	70%	3.8	N	Y	N
4	M	49	N	Y	N	1.0 (MRI)	N	Y	30%	2.8	N	Y	N
5	F	30	Y	N	N	1.7 (MRI)	Y	Y	GTR	1.5	N	Y	N
6	F	48	N	N	Y	1.6 (MRI)	N	Y	GTR	1.4	N	Y	N
7	M	76	N	N	Y	1.6 (MRI)	N	Y	GTR	0.5	N	Y	N
8	F	30	Y	Y	N	1.8 (MRI)	Y	Y	GTR	–	–	–	–
9	M	62	N	N	Y	1.3 (MRI)	Y	Y	50%	N/A	N/A	N/A	Y
10^a^	F	59	Y	N	N	2.0 (MRI)	Y	Y	60%	10.1	Y	Y	Y
11^a^	M	77	Y	Y	N	2.7 (CT)	Y	Y	85%	3.8	N	Y	N
	1:2: 1 (M:F)	53.0 ± 16.2	54.6%	45.5%	27.3%	2.04 ± 0.8	72.7%	100%	79.0% ± 25.3%	3.7 ± 3.3	90%	100%	20.0%

Values are presented as percentages or mean ± standard deviation. Abbreviations:; *CT* computerized tomography, *DI* diabetes insipidus, *F* female, *GTR* gross total resection, *M* male, *MRI* magnetic resonance imaging, *N* no, *N/A* not applicable, *Y* yes; *---* data not available

^a^Atypical pituicytoma

### Radiologic characteristics

All patients in the cohort had pre-operative imaging (either CT or magnetic resonance imaging [MRI] scans) and the findings are included in Table 1. The average tumor size was 2.04 ± 0.8 cm (range 1.0 to 3.5 cm) by imaging. 100% of the tumors involved the suprasellar region, and 73% also involved the sella. A preoperative radiologic differential was given for 9 patients. Meningioma, pituitary adenoma, and craniopharyngioma were the most common tumors listed in the differential diagnosis (44% of cases each), followed by glioma (33% of cases), and infectious (11% of cases). In four of the cases, the pre-operative radiologic differential included multiple entities. For the other five cases, a single the pre-operative radiologic diagnosis was given (pituitary adenoma, 4 cases; meningioma, 1 case). A specific diagnosis of pituicytoma was mentioned in the radiologic differential for one tumor (tumor #4, 11% of cases). Post-operative imaging was available for ten of the eleven patients. On average, 79.0% ± 25.3% (range 30% to ~ 100%) of the tumor was resected; four patients had gross total resections. Only one patient (#10) had radiographic evidence of progression or recurrence.

### Histologic characteristics

**Fig. 1:**
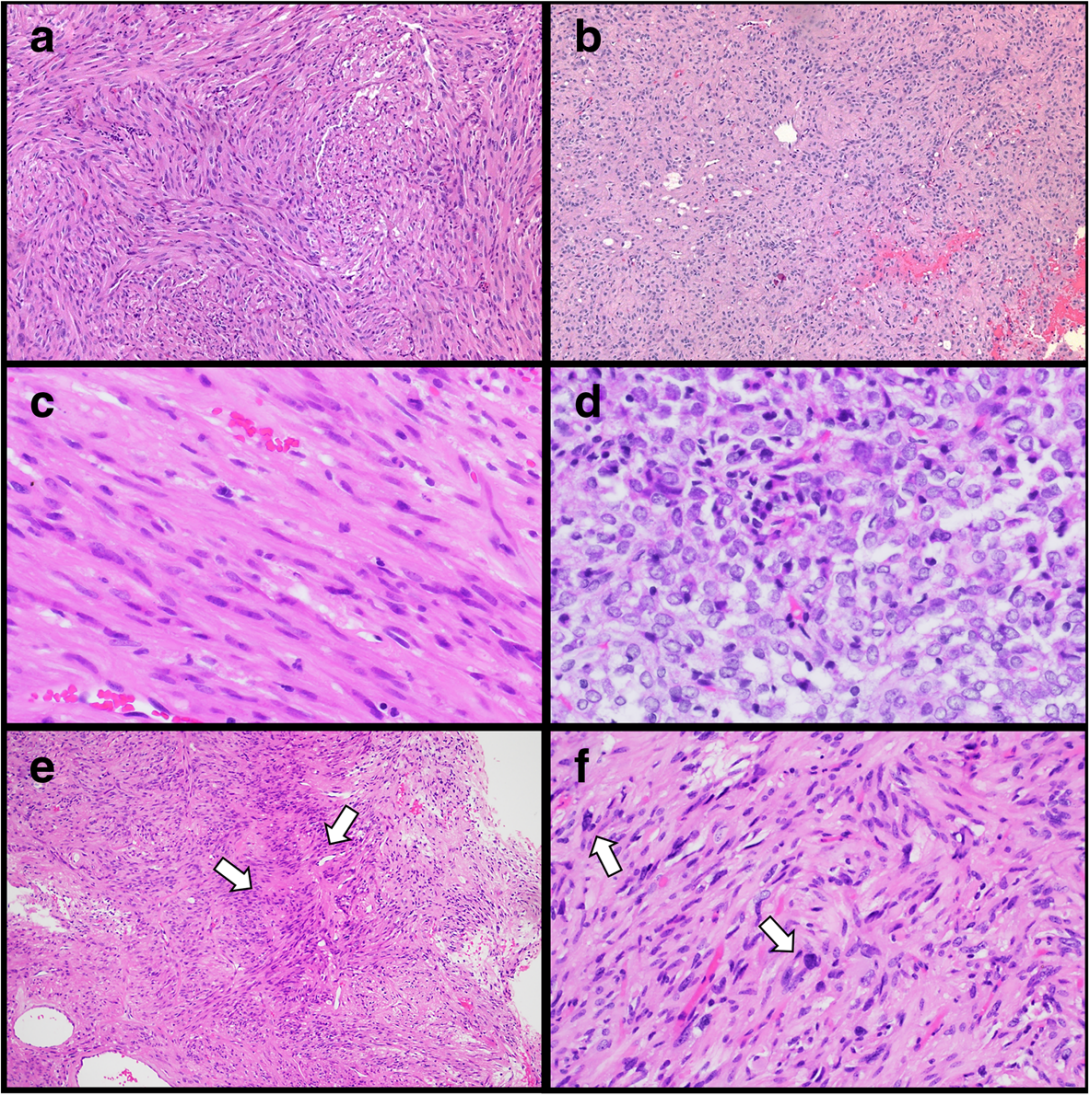
Histologic features of pituicytomas. Morphology varied across tumors, and included both fascicular and sheet-like growth patterns (**a** and **b**, respectively). Nuclei ranged from spindled to epithelioid (**c** and **d**, respectively). Vague nuclear palisade structures were present (**e**, highlighted by arrows). While pleomorphism was mild, some tumors showed focal areas of ancient-type change (**f**, highlighted by arrows). All images stained with hematoxylin and eosin. **a**, **b** and **e** at 100x; **c** and **d** at 200x; **f** at 400x

Pituicytomas showed morphologic variability. Some tumors grew in fascicles while others showed a vaguely lobulated or sheet-like growth pattern (Fig. 1a and b, respectively). Rarely, tumors exhibited vague nuclear palisades (Fig. 1e). Tumor cells had a moderate amount of eosinophilic cytoplasm, a consistent feature across all tumors; however, some tumors had a glial-like background while others had sharper cell borders and an epithelioid appearance. Granular cytoplasm was not present in any of these tumors. The tumor nuclei ranged from spindled to rounded (Fig. 1c and d, respectively), sometimes within the same tumor. Small nucleoli were present. In the non-atypical pituicytomas, there was minimal pleomorphism though few tumors showed changes resembling the “ancient change” seen in schwannomas (Fig. 1f). Numerous vessels of varying diameter were often present within the tumors. Mitotic activity was very sparse in non-atypical tumors (< 1 mitosis per 10 high power fields) and necrosis was not seen. Normal tissues were not present in any of the resections so the tumors could not be evaluated for the presence or absence of invasion or infiltration. Eosinophilic granular bodies and Rosenthal fibers were not identified in any of the fourteen specimens.

### Immunohistochemical characteristics

**Fig. 2:**
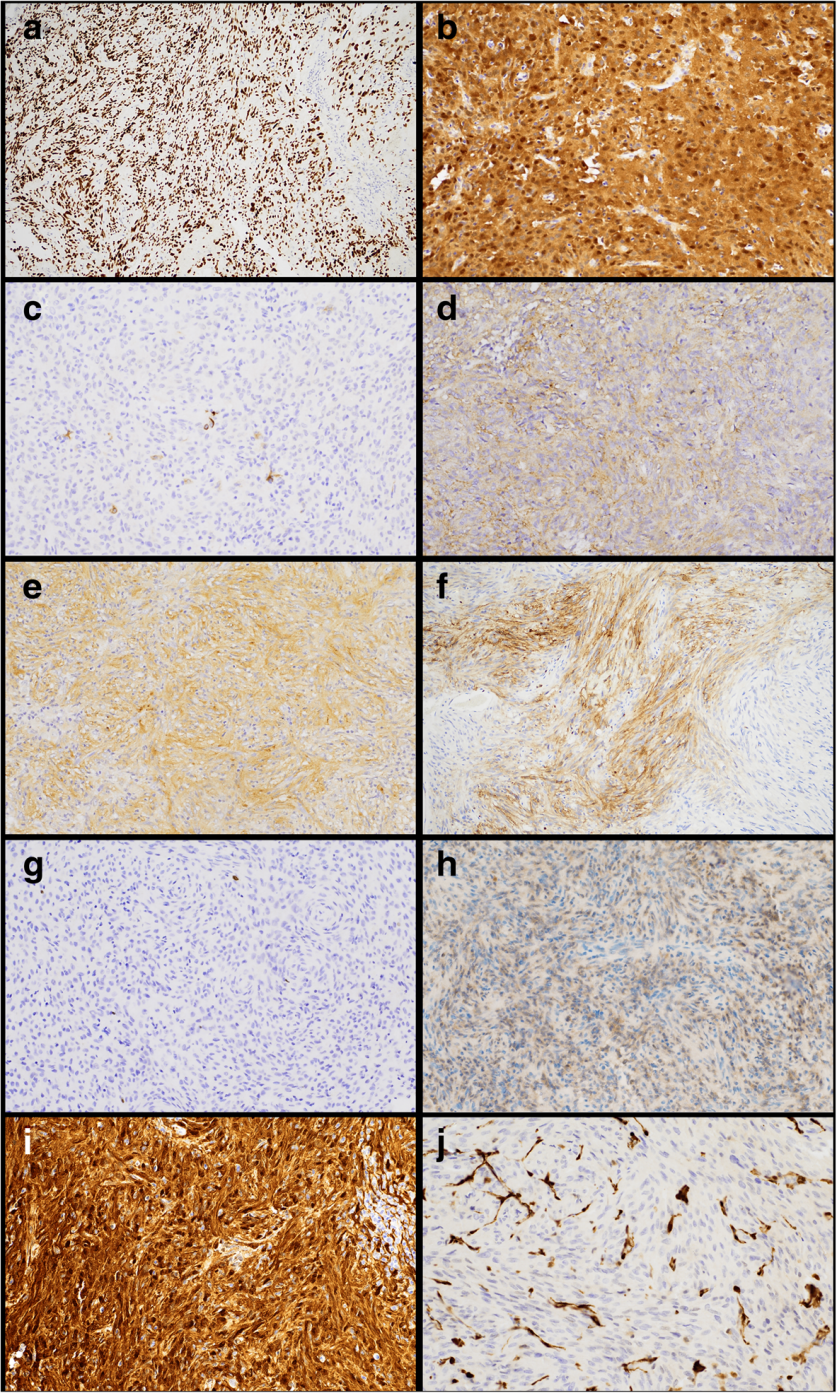
Immunohistochemical features of pituicytomas. All tumors showed strong and diffuse nuclear positivity for TTF-1 (**a**). A subset of tumors were positive for S100, GFAP (focal staining showed), EMA, synaptophysin and SSTR2A (**b**, **c**, **d**, **e** and **f**, respectively). The proliferation index by Ki-67 was low for non-atypical pituicytomas (**g**). One tumor (patient #1) was positive for BRAF V600E (**h**). The majority of tumors were strongly positive for pERK (**i**) though one was negative (**j**). **a** and **f** at 100x; **b**, **c**, **d**, **e**, **g**, **h**, **i** and **j** at 200x.

The immunohistochemical staining profile for all patients is summarized in Table 2. All tumors showed strong and diffuse nuclear positivity for TTF-1 (Fig. 2a). Eight of nine tumors stained for S100 were positive (89%, Fig. 2b). 50% of stained tumors showed at least focal positivity for GFAP (Fig. 2c). One third of tumors were positive for EMA (Fig. 2d), and one third showed synaptophysin positivity (Fig. 2e). SSTR2A was focally positive in 20% of stained tumors (Fig. 2f). The proliferation index by Ki-67 immunostain was low, ranging from < 1 to 4% in non-atypical pituicytomas (Fig. 2g) with a mean of 1.9% ± 1.2. Immunohistochemical findings did not correlate with various tumor morphologies (e.g. spindled vs epithelioid). Additionally, positive or negative immunostaining for one marker was not predictive of the staining pattern for another marker. For patient #10, whose tumor was resected on four separate occasions, immunohistochemical staining was consistent across all resections.

**Table 2:** Immunohistochemical characteristics of pituicytomas

Patient	TTF-1	EMA	S100	GFAP	Synapto-physin	Ki-67	SSTR2A	pERK	BRAF V600E
1	+	–	+	Focal +	+	3%	–	+	+
2	+	–	+	–	–	< 1%	–	+	–
3	+	+	+	–	NP	4%	Focal +	–	–
4	+	–	NP	Focal +	Focal +	NP	–	+	–
5	+	NP	NP	+	NP	< 1%	–	+	NP
6	+	+	+	+	NP	< 1%	NP	+	NP
7	+	Focal +	+	Focal +	NP	1–3%	–	+	NP
8	+	NP	+	NP	NP	NP	–	+	–
9	+	–	Focal +	–	NP	< 1%	–	+	NP
10 (1)^a^	+	–	–	NP	NP	5%	NP	NP	NP
10 (2)^a^	+	–	–	–	NP	10%	NP	NP	NP
10 (3)^a^	+	NP	NP	–	NP	NP	NP	NP	–
10 (4)^a^	+	–	NP	–	NP	10%	Focal +	+	–
11^a^	+	–	+	–	+	10%	–	+	–
% Positive	100.0%	33.3%	88.9%	50.0%	33.3%		20.0%	90.9%	14.3%

Patient 10 had four separate resections as indicated by the parentheses. Abbreviations: *+* positive; *-* negative, *NP* not performed

^a^atypical pituicytoma

### Molecular findings

One tumor was sequenced on our institution’s Solid Tumor Sequencing Panel and another was sequenced on both the Solid Tumor and Fusion Transcript Panels as part of clinical care. Additionally, DNA was extracted from formalin-fixed, paraffin-embedded (FFPE) tissue for eight tumors as part of this research study to be assayed on the Solid Tumor Sequencing Panel. There was insufficient material available for sequencing in the remaining four cases. Sufficient DNA was extracted from a total of three tumors (including the two sequenced for clinical purposes) and sufficient RNA was extracted from one tumor. For the other seven tumors, the extracted DNA did not meet quality standards to be run on the Solid Tumor Sequencing Panel. Table 3 summarizes the NGS findings for each tumor. For all sequenced tumors, the estimated tumor percentage was > 50% which was supported by immunohistochemical staining for TTF-1.

**Table 3:** Next generation sequencing of pituicytomas

Patient	Sequencing attempted	DNA adequate	Disease-Associated Variants^b^		Variants of Uncertain Significance^b^		RNA adequate	Abnormal fusion Transcripts
1	Y	Y	*BRAF* c.1799 T > A p.(V600E)	VAF 7%	*ARID2* c.1672C > T p.(R558C) *FLT3* c.2546G > A p.(R849H)	VAF 4% VAF 3%	N/A	N/A
			*NF1* c.7079_ 7082delTTAT p.(F2360Wfs*35)	VAF 13%	*IGF1R* c.3897C > G p.(N1298K) *MSH6* c.1157C > G p.(P386R)	VAF 50% VAF 48%		
			*TSC1* c.2074C > T p.(R692*)	VAF 2%	*PBRM1* c.2504G > A p.(R850H); *RNF43* c.1114C > T p.(P372S)	VAF 4% VAF 55%		
2	Y	N	N/A		N/A		N/A	N/A
3	Y	N	N/A		N/A		N/A	N/A
4	Y	N	N/A		N/A		N/A	N/A
5	Y	N	N/A		N/A		N/A	N/A
6	Y	N	N/A		N/A		N/A	N/A
7	Y	Y	*HRAS* c.182A > G p.(Q61R)	VAF 26%	*KIT* c.287C > T p.(T96 M) *PTCH1* c.3617G > A p.(R1206H)	VAF 38% VAF 49%	Y	None
8	N	N/A	N/A		N/A		N/A	N/A
9	N	N/A	N/A		N/A		N/A	N/A
10 (1)^a^	N	N/A	N/A		N/A		N/A	N/A
10 (2)^a^	N	N/A	N/A		N/A		N/A	N/A
10 (3)^a^	Y	N	N/A		N/A		N/A	N/A
10 (4)^a^	Y	Y	None		*KDR* c.3937G > A p.(D1313N)	VAF 47%	N/A	N/A
11^a^	Y	N	N/A		N/A		N/A	N/A

Patient 10 had four separate resections as indicated by the parentheses. Abbreviations: *N* no, *N/A* not applicable, *VAF* variant allele fraction, *Y* yes

^a^Atypical puticytoma

^b^VAF may vary within +/− 10% of the actual value, due to stochastic error in the NGS assay. Additionally, tissue heterogeneity and technical artifact such as primer bias may affect the VAF. Therefore, the actual VAF in these tumors may be higher than reported

One tumor (patient #7) was found to have a disease associated variant *HRAS* c.182A > G p.(Q61R) as well as variants of uncertain significance in *KIT* c.287C > T p.(T96 M) and *PTCH1* c.3617G > A p.(R1206H). A second tumor (patient #1) was found to have three disease associated variants: *BRAF* c.1799 T > A p.(V600E), *NF1* c.7079_7082delTTAT p.(F2360Wfs*35), and *TSC1* c.2074C > T p.(R692*). This same tumor also had six variants of uncertain significance: *ARID2* c.1672C > T p.(R558C), *FLT3* c.2546G > A p.(R849H), *IGF1R* c.3897C > G p.(N1298K), *MSH6* c.1157C > G p.(P386R), *PBRM1* c.2504G > A p.(R850H), and *RNF43* c.1114C > T p.(P372S). Finally, the third tumor, #10 (4th resection), did not have any disease associated variants but was found to have a variant of uncertain significance in *KDR* c.3937G > A p.(D1313N).

For the one tumor (patient #7) successfully sequenced on the Fusion Transcript Panel, no abnormal fusion transcripts were detected.

### Immunohistochemical staining for BRAF V600E and Phosphorlyated ERK

Given the results of the NGS and our hypothesis that alterations in the MAPK pathway may be seen in pituicytomas and that these alterations may be detectable at the protein level by immunohistochemical techniques, staining for BRAF V600E and pERK was performed on cases with adequate tissue. Ten of eleven tumors stained were strongly and diffusely positive (both nuclear and cytoplasmic staining) for pERK (90.9%, Fig. 2i and j), including those with the BRAF and HRAS mutations. One tumor (patient #1) showed patchy positive staining for BRAF V600E (Fig. 2h), consistent with the *BRAF* c.1799 T > A p.(V600E) mutation detected on NGS.

Five non-neoplastic pituitary glands were also stained for pERK (Additional file 1: Figure S1). Pituicytes did not show strong cytoplasmic or nuclear staining for pERK though in two cases, weak to moderate cytoplasmic staining was present (Additional file 1: Figure S1d and 1e, compare to Additional file 1: Figure S2f and i). Only rare cells in the anterior pituitary were positive for pERK (Additional file 1: Figure S1b).

### Atypical pituicytomas

**Fig. 3:**
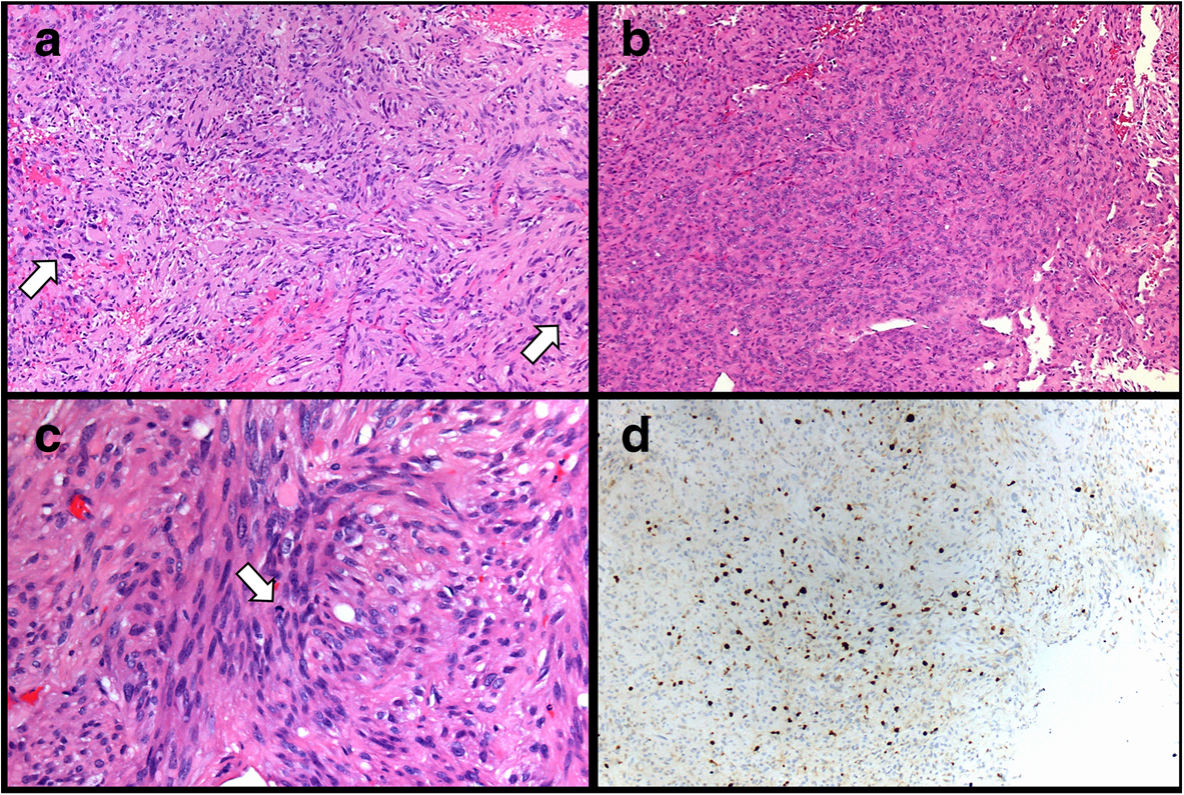
Histologic and Immunohistochemical features of atypical pituicytomas. Tumors showed high cellularity and noticeable atypia (**a** and **b**, arrows in **a** highlighting regions with atypical nuclei). Mitotic figures were present (**c**, arrow pointing to mitosis). Atypical pituicytomas had an elevated Ki-67 proliferation index (**d**). **a**, **b** and **c** stained with hematoxylin and eosin. **a**, **b** and **d** at 100x; **c** at 200x

Two patients in the cohort had pituicytomas that were classified as “atypical” on the basis of histology. These tumors showed increased cellularity, atypia and mitoses (up to 4 mitoses per 10 high power fields, Fig. 3a-c). Tumor #10(4), resection of which followed treatment with radiation, also showed areas of necrosis and radiation-type nuclear atypia. While these tumors did not show significant difference in their immunohistochemical profile from non-atypical pituicytomas, they did have higher Ki-67 proliferation indexes ranging from 5 to 10% (Table 2, Fig. 3d). Clinically, one patient’s tumor recurred at 2 and 3 years following the initial resections (#10). Sequencing performed on the patient’s 4th resection revealed a variant of uncertain significance in *KDR* (Table 3). This patient was treated with chemotherapy and radiation and is now deceased secondary to complications of radiation therapy. For the second patient whose pituicytoma demonstrated atypical features (#11), head imaging performed two years after surgery showed residual enhancing mass within the sella, slightly increased in size from prior studies. The patient was lost to follow-up approximately 4 years after surgery.

### Radiologic differential

Given the possibility of targeted therapy, the question arises whether the diagnosis of pituicytoma may be suspected or definitively made pre-operatively. The diagnosis is often not considered, due to the rarity of the tumor and the fact that the known imaging features overlap significantly with those of pituitary adenoma. To see whether an experienced neuroradiologist might be able to differentiate pituicytoma from other sellar/suprasellar neoplasms, one neuroradiologist (IMN) reviewed a mixed cohort including ten pituicytomas, seven pituitary adenomas and three craniopharyngiomas. The radiologist was blinded to the diagnoses, but given the information that 50% of the cases were pituicytomas. The correct diagnosis of pituicytoma was rendered in 7/10 cases of pituicytomas. All three pituicytomas that were misdiagnosed were called adenoma. In two of these three cases, the imaging was noted to be suboptimal. One adenoma was misdiagnosed as a pituicytoma; all other adenomas and all craniopharyngiomas were correctly diagnosed.

## Discussion

Pituicytomas are rare, sellar and/or suprasellar tumors arising from the pituicytes of the neurohypophysis. Due to the rarity of this tumor, most of the literature exists as case reports and small case series. Currently, just over 80 cases of pituicytoma have been published [23]. Our cohort of fourteen pituicytoma resections from eleven patients, to our knowledge, matches the size of the largest single-institution study to date. Our cohort also includes two patients with atypical pituicytomas.

Pituicytomas classically occur in adult patients (mean age of approximately 50) with nearly two thirds of patients diagnosed between ages 40 and 60; a slight male predominance has been reported [5, 10, 14]. Rare pediatric cases exist in children as young as 7 [27]. Our findings mirror those in the literature; the average age at presentation in our cohort was 53 and our cohort showed a slight male predominance. The majority of patients present with visual disturbance and/or headache followed by fatigue and endocrine abnormalities [3, 6] and similar symptoms were present in this cohort. Pituicytomas are very vascular tumors, and there can be significant intra-operative bleeding [2, 5, 12, 18]. Reported surgical complications following pituicytoma resection include hypopituitarism, diabetes insipidus, and vision loss [6, 8, 27, 28]. All surviving patients in our study had persistent endocrine abnormalities as complications of surgical resection.

On imaging, pituicytomas are uniformly contrast-enhancing masses, and the reported radiologic findings are nonspecific and show overlap with other tumors of the sellar region including pituitary adenomas, craniopharyngioma, and meningiomas [1, 9, 24, 26, 27]. The pre-operative radiologic differentials for the tumors in this study often listed meningioma, pituitary adenoma and craniopharyngioma. Pituicytoma was listed in the differential for only one patient. Other studies similarly found that pituicytomas are nearly always misdiagnosed prior to surgery [2, 12]. The misdiagnosis is most likely due to the rarity of the tumor; when actively considered in the differential given a known prevalence in the cohort, the correct tumor diagnosis was given in 16/20 cases. Giving radiological consideration for the diagnosis of pituicytoma in every sellar/suprasellar tumor case in clinical practice will lead to a high rate of misdiagnoses for pituicytoma, given the extremely low prevalence. The imaging diagnosis of pituicytoma was based on the appearance of the tumor originating in a suprasellar rather than sellar location, unless it had a cystic component compatible with craniopharyngioma. Although the diagnosis may be suspected pre-operatively, histologic examination remains essential for accurate diagnosis of this tumor.

Pituicytomas have been reported to show a range of histologic characteristics [1, 7, 14, 19, 23, 24] similar to those described in this study. One explanation for this variation may be differences in normal pituicyte (putative cell of origin) morphology [22]. For non-atypical tumors, common features include rounded to spindled nuclei, eosinophilic cytoplasm, small nucleoli, a lack of nuclear pleomorphism, absent to minimal mitotic activity, and no invasion of normal structures. All tumors in this study were positive for TTF-1. Given the variability in staining for other markers including GFAP, EMA, S100, SSTR2A and synaptophysin shown in this study and others [1, 7, 10, 15], TTF-1 is the most reliable immunostain for diagnosis. The use of this immunostain is particularly important given the histologic and immunohistochemical overlap with other tumors of the sellar region (e.g. meningiomas and positive EMA staining). For non-atypical tumors, Ki-67 proliferative indexes are low (< 1–4% in this study and < 3% in the literature; [1, 10]).

Prior attempts to elucidate molecular signatures of pituicytomas were largely unrevealing. To date, all tumors tested were negative for *IDH* mutations and *BRAF* alterations [15]. However, an *HRAS* mutation was seen in a spindle cell oncocytoma [16], which is likely a morphologic variant of pituicytoma [10, 11, 15]. In our study, NGS was able to be performed on three tumors. As seen in prior studies, no *IDH* mutations or *BRAF* fusions were detected in our cohort. However, one tumor harbored a disease-associated variant in *HRAS*, and a *BRAF* p.(V600E) disease-associated variant was detected in another. The third tumor had a variant of uncertain significance in *KDR.* Interestingly, *HRAS*, *BRAF* and *KDR* all encode proteins involved in the MAPK pathway, which regulates cell division in response to growth factors. Mutations in this pathway are seen in many cancer types, including tumors of the central nervous system [4, 17, 20]. Selective MAPK pathway inhibitors have been developed for the treatment of various tumors with MAPK pathway alterations [2, 25]. Given the morbidity associated with resecting pituicytomas, and potential complications of radiation therapy, these targeted therapies may present a novel therapeutic option for treatment of pituicytomas, although tissue sampling remains necessary for diagnosis and molecular testing at this time.

While alterations in genes involved in the MAPK pathway were seen in all three successfully sequenced tumors, NGS was unable to be performed in seven tumors due to insufficient quality or quantity of DNA. All cases sequenced for clinical reasons were successful, and in these instances, FFPE tissues were cut for molecular testing shortly after surgery. In cases where insufficient DNA was extracted from FFPE, this is likely due to limited quantities of tissue within the blocks and/or the age of the tissue. Although sequencing could not be performed for all cases, pERK staining was strongly and diffusely positive in all but one case. Positive staining was seen in all tumors with variants in MAPK pathway genes, but further investigation is required to determine whether this immunostain is a sensitive and specific marker of alteration in the MAPK pathway in pituicytomas. Overall, these findings support that alterations in MAPK signaling may be contributing to pituicytoma tumorigenesis.

Similar to our findings in pituicytomas, other tumor types associated with MAPK alterations show a number of different mutations. For example, melanomas and colorectal carcinomas may exhibit mutations in RAS, BRAF and MEK [2]. Though purely speculative in the context of pituicytomas, it is possible that pituicytes are highly sensitive to alterations in MAPK signaling and any number of perturbations leading to increased phosphorylation of ERK may contribute to tumorigenesis. Very rarely, two mutations within the MAPK pathway have been reported in a single tumor. It has been proposed that tumor heterogeneity allows for multiple alterations within this pathway to be present within an individual tumor and provides evidence that tumors demonstrate clonal evolution and plasticity over time [2].

While pituicytomas are histologically compatible with WHO grade I and generally considered to be indolent, these tumors have been shown to recur [1, 2, 12]. One tumor in our study recurred 2 and 3 years following the initial resections despite chemotherapy and radiation (patient #10). This tumor was shown to have an elevated Ki-67 proliferation index. Another tumor (patient #11) also had an elevated Ki-67 proliferation index. For both tumors, increased cellularity, atypia and mitoses were also present. These tumors were diagnosed as atypical given these findings not classically seen in pituicytomas. One other pituicytoma with increased Ki-67 proliferation index, increased mitoses and moderate nuclear pleomorphism has been described in the literature (Hagel et al., 2017, case #22 [10]). The biologic significance of these histologic and immunohistochemical findings is still uncertain, but histologically atypical pituicytomas may warrant close clinical and radiologic follow-up.

## Conclusion

In conclusion, pituicytoma represents a rare tumor of the sella and suprasellar region. They show varied morphologies and immunohistochemical profiles but are consistently TTF-1 positive. Resection of pituicytomas is associated with persistent, significant endocrine abnormalities. Using NGS and immunohistochemistry, we show an association of MAPK activation in pituicytomas, a pathway potentially treatable with MAPK inhibitors. Establishing these molecular changes in pituicytomas may lay the groundwork for investigating the development of targeted therapy for pituicytomas.

## Additional file

Additional file 1: **Figure S1.** pERK staining in non-neoplastic pituitary glands. Representative image of non-tumoral anterior pituitary on H&E stain (**a**). The anterior pituitary was overall negative for pERK with rare cells showing strong positivity (**b**). Representative image of non-tumoral neurohyophysis on H&E stain (**c**). In three of five specimens, the pituicytes showed no appreciable staining for pERK though staining was present in vessels (**d**). In two of five specimens, weak to moderate cytoplasmic staining for pERK was seen though strong nuclear staining was only present in vessels (**e**). Representative example of pERK staining in a pituicytoma with strong nuclear and cytoplasmic staining (**f**). All images at 200x. (TIF 30271 kb)

## Abbreviations

CPD: Center for Personalized Diagnostics
FFPE: Formalin-fixed, paraffin-embedded
NGS: Next generation sequencing
p-ERK: Phosphorylated-ERK
SSTR2A: Somatostatin Receptor 2
TTF1: Thyroid transcription factor 1
VAF: Variant allele fraction
