# Supplementary figures and images for: Histologic, immunohistochemical, and molecular features of pituicytomas and atypical pituicytomas
Source: Acta Neuropathol Commun. 2019 May 2;7:69. doi: 10.1186/s40478-019-0722-6 (PMC6498683; doi:10.1186/s40478-019-0722-6)

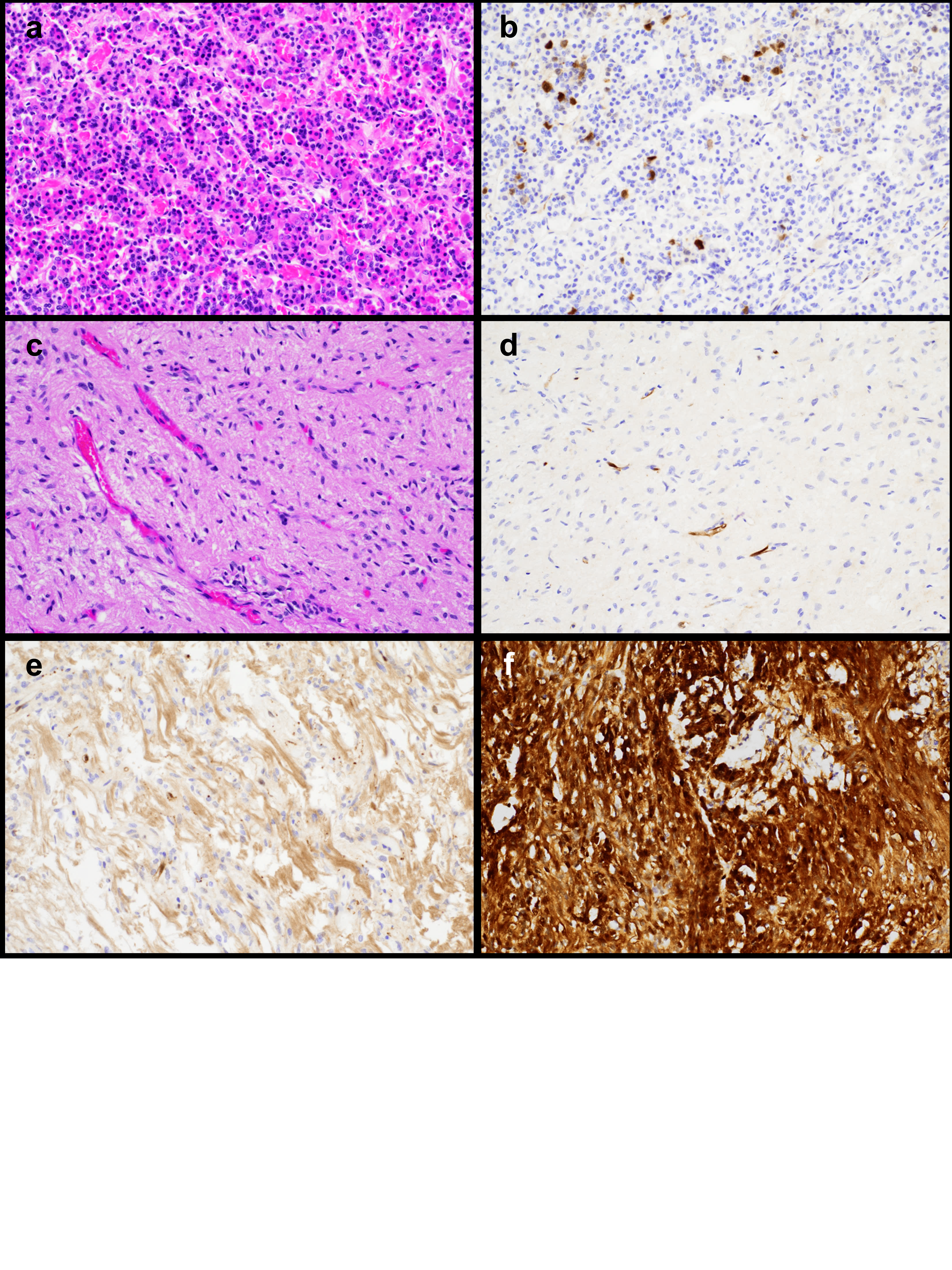

Supplement: Supplementary file 1 — Figure S1. pERK staining in non-neoplastic pituitary glands. Representative image of non-tumoral anterior pituitary on H&E stain (a). The anterior pituitary was overall negative for pERK with rare cells showing strong positivity (b). Representative image of non-tumoral neurohyophysis on H&E stain (c). In three of five specimens, the pituicytes showed no appreciable staining for pERK though staining was present in vessels (d). In two of five specimens, weak to moderate cytoplasmic staining for pERK was seen though strong nuclear staining was only present in vessels (e). Representative example of pERK staining in a pituicytoma with strong nuclear and cytoplasmic staining (f). All images at 200x. (TIF 30271 kb) [file 40478_2019_722_MOESM1_ESM.tif]
